# Mini-implant selection protocol applied to MARPE

**DOI:** 10.1590/2177-6709.23.5.093-101.sar

**Published:** 2018

**Authors:** Lincoln Issamu Nojima, Matilde da Cunha Gonçalves Nojima, Amanda Carneiro da Cunha, Natan Oliveira Guss, Eduardo Franzotti Sant’Anna

**Affiliations:** 1 Universidade Federal do Rio de Janeiro, Department of Pediatric Dentistry and Orthodontics (Rio de Janeiro/RJ, Brazil).; 2 Universidade Federal do Rio de Janeiro, Post-graduation Program in Orthodontics (Rio de Janeiro/RJ, Brazil).

**Keywords:** Palatal expansion technique, Orthodontic anchorage procedures, Malocclusion, Transverse maxillary atresia

## Abstract

**Introduction:**

Rapid maxillary expansion (RME) is the therapy of choice to correct skeletal transverse dimension in children and adolescents, associating orthopedic and dental effects. In an attempt to prevent the undesirable dentoalveolar effects and optimize the potential of skeletal expansion in individuals in advanced stages of skeletal maturation, the miniscrew-assisted rapid palatal expander (MARPE) was proposed by Lee et al. in 2010.

**Objective:**

This paper presents a systematized protocol for selection of miniscrews indicated for MARPE, by the evaluation of cone-beam computed tomographies (CBCT). Variables related with the bone and soft tissue thicknesses at the palatal regions of interest, as well as in relation to the fixation rings of miniscrews of the palatal expander are analyzed and discussed to provide better performance in the clinical practice.

## Resumo

**Introdução:**

a expansão rápida da maxila (ERM) é a terapia de escolha para a correção da dimensão transversa esquelética em crianças e adolescentes, associando-se efeitos ortopédicos e dentários. Com a finalidade de prevenir os efeitos dentoalveolares indesejáveis e otimizar o potencial de expansão esquelética em indivíduos com estágios avançados de maturação esquelética, a técnica de expansão rápida da maxila assistida por mini-implantes (MARPE) foi proposta por Lee e colaboradores em 2010.

**Objetivo:**

o presente estudo apresenta um protocolo sistematizado para seleção de mini-implantes indicados para a MARPE, mediante avaliação de imagens de tomografia computadorizada de feixe cônico (TCFC). Variáveis relacionadas à espessura de tecido ósseo e tecido mole nas regiões de interesse do palato, bem como em relação ao anel de fixação dos mini-implantes do parafuso expansor, são analisadas e discutidas para proporcionar melhor desempenho na prática clínica.

## INTRODUCTION

Data of the last national epidemiological survey of oral health, the SB Brasil 2010 (National Oral Health Investigation), indicated the prevalence of posterior crossbite in 21.9% of Brazilian individuals at the age of 5 years.^1^ However, besides posterior crossbites, the presence of crowding, alterations in dental axial inclinations, wide buccal corridors and some Class II and Class III sagittal malocclusions^2^ may have the transverse maxillary deficiency as underlying etiologic factor, which is often not diagnosed in the daily clinical practice, indicating that the actual prevalence is possibly higher than currently reported rates.^3^

The rapid maxillary expansion (RME) is the procedure of choice to reestablish the skeletal transverse dimension in children and adolescents, by the association of orthopedic and dental effects,^4^ ^-^ ^6^ consisting of the biomechanical principle of separating the two maxillary halves by remodeling of the midpalatal suture and intermaxillary sutures.^4^ ^,^ ^6^ ^,^ ^7^

Based on a biological perspective, the treatment prognosis of adult patients with RME is doubtful, due to the increased interdigitation of maxillary sutures and rigidity of adjacent structures, such as the zygomaticomaxillary pillar.^8^ Embryonic aspects of the midpalatal suture formation indicate the presence of some obliteration, primarily located at its posterior region, with different degrees of obliteration along its pathway.^9^ Also, the high complexity level of the articulation between palatal bones with the sphenoid bone, posteriorly; and the maxilla, anteriorly, assign considerable resistance to displacement of the posterior maxillary region, both in vertical and horizontal directions.^10^ ^,^ ^11^ Consequently, root resorptions, damage to periodontal tissues,^12^ ^-^ ^14^ technique failure or limitations,^15^ questionable stability over time,^16^ edemas and soft tissue lesions^17^ are associated with RME in individuals who reached skeletal maturity. 

The miniscrew-assisted rapid palatal expansion (MARPE) was proposed by Lee et al^18^ in 2010, aiming to solve the undesirable dentoalveolar effects and optimize the potential of skeletal expansion in individuals in advanced stages of skeletal maturation. Effective separation of the midpalatal suture was observed in an adult patient with mild buccal inclination of maxillary molars.^18^

There is increasing interest in the scientific literature in this field, with recent publications of case reports illustrating the different variations of MARPE devices and its expansion protocols;^19^ ^-^ ^21^ besides a finite element study on the role of miniscrews in the distribution of forces in MARPE devices.^22^ A retrospective clinical study conducted on young adults submitted to MARPE revealed opening of the midpalatal suture in 86.9% of cases, with stable outcomes at 30-month follow-up, by evaluation of posteroanterior cephalograms.^23^ Cone-beam computed tomographies (CBCT) revealed significant increases in dentoalveolar and skeletal dimensions in young adults treated with MARPE and followed for one year after expansion.^24^ Therefore, the MARPE is a clinically effective and stable approach for non-surgical correction of transverse discrepancy in adult patients. Thus, this paper aimed to propose a systematized protocol for selection of miniscrews indicated for MARPE, with the aid of CBCT scans, which present as an effective diagnostic resource in Orthodontics.

## UTILIZATION OF CBCT TO GUIDE MINI-IMPLANTS INSERTION IN MARPE

The great advantage of cone-beam computed tomographies compared to bidimensional dental radiographs is the absence of superimposition of anatomical structures. Besides the visualization of multiplanar sections in axial, coronal and sagittal planes, the images provide accuracy of approximately 0.2 mm, which is adequate for clinical applicable measurements. The digital measurement tools provided in softwares for DICOM visualization allow measurement of any distance, area or volume in acquired images. These characteristics are important to evaluate the bone thickness at areas adjacent to the midpalatal suture, where miniscrews are inserted according to the planning for MARPE.

The MARPE^18^ technique comprises the insertion of four miniscrews adjacent to the midpalatal suture, being two mesial and two distal to the expanding screw. Among the anatomical characteristics at this area, the mean thickness of bone present in the regions mesial and distal to the expanding screw varies, respectively, from 3.77 to 3.88 mm and from 2.33 to 2.44 mm.^25^ Similarly, the soft tissues present variation in thickness of 2.6 to 2.8 mm and 1.75 to 1.82 mm, respectively, at the regions mesial and distal to the expanding screw.^25^ This variability in bone and soft tissue thickness, associated with the height of the fixation ring of the expander miniscrew and its distance in relation to the soft tissue, impair the appropriate selection of the miniscrew length. 

**Figure 1:**
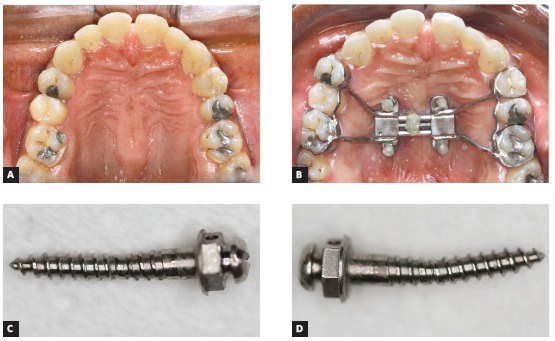
A, B) Clinical photographs in occlusal view, before and after expansion, respectively. C, D) Mesial right and mesial left miniscrews used as anchorage for MARPE, after its removal from the oral cavity.

Concerning the type of insertion of miniscrews, the bicortical insertion is recommended, particularly with anchorage in internal cortical plates of the palate and nasal fossa. The fixation in both cortical plates is fundamental to aid the anchorage during expansion and to surpass the resistance of maxillary bones to separation. When the monocortical insertion of miniscrews is used in individuals with thick suture or with great resistance to maxillary separation, distortions or folds may occur in the temporary anchorage device during activation of the expanding screw (Fig 1). Therefore, a correct selection of miniscrew length by analysis of bone tissue thickness and height of midpalatal suture, assessed by CBCT examination, is relevant for the success of MARPE.

## PROTOCOL FOR SELECTION OF MINI-IMPLANTS LENGTH IN MARPE

To select the length of miniscrews to be used in the MARPE technique, we suggest the following steps.

### 1) Achievement of dental casts

**Figure 2:**
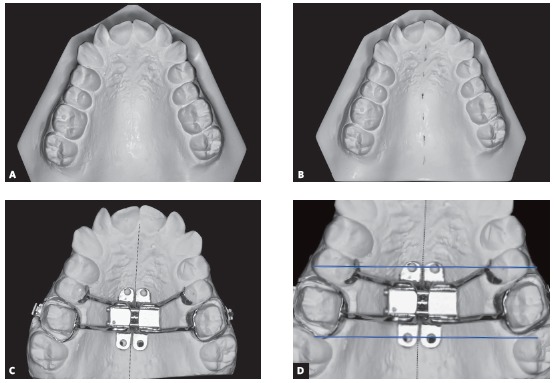
A) Initial dental cast. B) Delineation of dotted line referring to the midpalatal suture. C) Positioning of expanding screw coinciding with the midpalatal suture line. D) Delineation of reference lines (in blue), transverse to the midpalatal suture, passing through the center of miniscrews fixation rings mesial and distal to the expanding screw.

The working dental cast is obtained after transfer impression with bands positioned on teeth #16 and #26. Initially, the line referring to the midpalatal suture is traced, which delineates the expanding screw in relation to its transverse position (Figs 2A and 2B). The sites of miniscrew insertion are selected with the expanding screw pre-positioned on the dental cast. In anteroposterior direction, as reference, the expanding screw may be positioned at the level of permanent first molars (Fig 2C). Two lines transverse to the midpalatal suture are traced, in mesial and distal direction to the expanding screw, passing through the center of fixation rings of miniscrews up to the occlusal surface of teeth (Fig 2D). These lines are used as reference for the achievement of coronal tomographic slices and measurement of bone thickness at the selected anatomical regions. 

### 2) Selection of DICOM visualization software and maxilla orientation in CBCT images

The bone height is measured on DICOM files generated from CBCT exams. The area to be included in the tomography (field of view, FOV) should be selected by the orthodontist, e.g. maxilla or full face. However, the cortical plate of the nasal fossa and occlusal aspects of maxillary teeth should be considered the minimum area included on the tomographic acquisition. 

**Figure 3:**
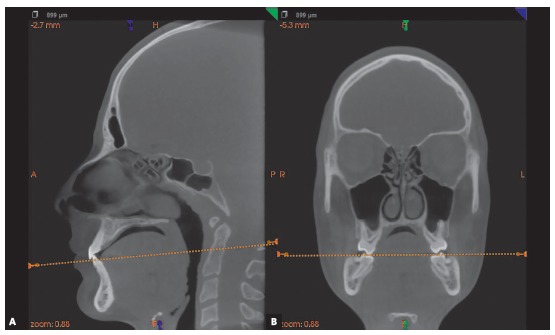
CBCT images illustrating the maxilla in multiplanar sections. A) Sagittal section. B) Coronal section. The axial section should coincide with the occlusal plane, delineated by the cusp tips of molars and incisal edges of maxillary central incisors.

DICOM visualization softwares, such as Dolphin (Dolphin Imaging & Management solutions, Chatsworth, CA, USA), ITK-SNAP (http://www.itksnap.org),^26^ 3DSlicer (http://www.slicer.org),^27^ CS 3D Imaging Software (Carestream Dental LLC Atlanta, GA, USA), InVivoDental (Anatomage, San Jose, CA, USA) or DentalSlice (DentalSlice, Brasília, DF, Brazil) may be indicated for that purpose. We suggest the CS 3D Imaging Software as an open source software for the evaluations, since it allows the orientation of multiplanar sections. To proceed with the maxilla orientation, the axial plane should coincide with the occlusal plane of maxillary teeth, i.e. the cusp tips of molars and incisal edges of maxillary central incisors (Fig 3).

### 3) Measurement of bone thickness on the coronal section of CBCT images

The following step comprises selection of the coronal sections that were predetermined on the dental casts, as indicated in Figure 2 sequence, following the identification of reference lines transverse to the midpalatal suture, passing through the fixation rings of miniscrews mesial and distal to the expanding screw. 

**Figure 4:**
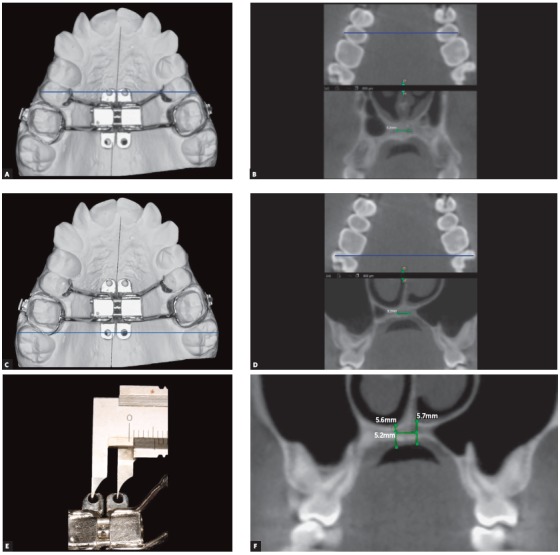
A) Dental cast illustrating the delineation of reference line (in blue) passing through the miniscrews fixation rings at the mesial region of the expanding screw, extending to the second premolars. B) CBCT images: on the axial section with the reference line (in blue), and on the coronal section evidencing the quantity of bone equidistant to the midpalatal suture, corresponding to the position of the reference line delineated on the axial section (in blue). C) Dental cast with delineation of reference line (in blue), passing through the miniscrews fixation rings at the distal region of the expanding screw and extending to the molars. D) CBCT images: on the axial section, with the reference line (in blue), and on the coronal section evidencing the quantity of bone equidistant to the midpalatal suture, corresponding to the level of the reference line delineated on the axial section (in blue). E) Measurement of width between the miniscrews fixation rings of the expanding screw, assessed with a caliper. F) CBCT images illustrating the measurement of bone and soft tissue height, on the coronal section corresponding to the distal region of the expanding screw.

At the mesial region of the expanding screw, as indicated on the dental cast, a plane is delineated crossing the fixation rings of miniscrews until beyond the respective teeth (Fig 4A). The same position is selected on the coronal section of the CBCT image (Fig 4B), taking as reference the teeth crossed by the plane defined on the dental cast. The same guidelines are followed for the fixation rings of miniscrews at the distal region of the expanding screw (Figs 4C and Fig 4D). Following, the width between fixation rings of miniscrews located on the expanding screw is measured with a caliper (Fig 4E). This measurement is transferred to the coronal section of the CBCT image, positioned on the central part of the bone, and equidistant to the midpalatal suture (Figs 4B and 4D). The miniscrew length indicated corresponds to the bone thickness measured on the coronal sections of CBCT images (Fig 4F). The soft tissue thickness may also be obtained at the same area of miniscrews insertion. For that purpose, the patient is instructed to avoid touching the palate with the tongue while tomography acquisition.

### 4) Evaluation of expander miniscrews fixation rings

**Figure 5:**
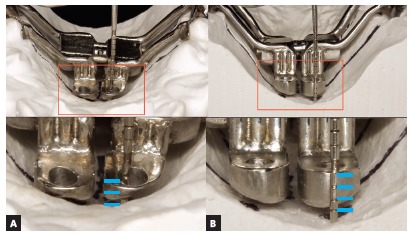
Measurement of height of fixation rings of anterior (A) and posterior miniscrews (B); and between them and the palatal surface assessed on the dental cast using a periodontal probe with millimeter marks.

To achieve the necessary length of miniscrews in MARPE, the height of expander miniscrews fixation rings to the expanding screw should be evaluated, as well as their distance from the palatal soft tissue surface (Fig 5), besides the bone and soft tissue measurements described in the previous step.

### 5) Selection of miniscrew 

**Figure 6:**
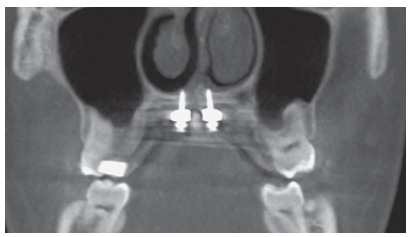
CBCT image on the coronal section at the distal region of the expanding screw, illustrating the bicortical insertion of miniscrews.

The total length of the miniscrew (MI) is represented by the variables: bone thickness (o), adding 1.0 to 2.0 mm, which is necessary for the miniscrew tip to surpass the cortical plate of the nasal fossa; soft tissue thickness (m); fixation ring thickness (a), and distance from the ring to the palatal surface (d). The equation employed to calculate the total miniscrew length is described, with the value in millimeters, as: MI= o + m + a + d + (1 or 2). The total MI length selected is related to the distance from its active tip to the base of the transmucosal collar (Fig 6). 

**Figure 7:**
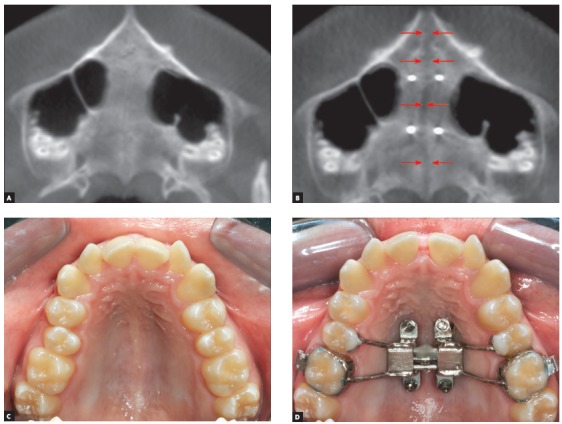
A, B) CBCT images on the axial section, before and after MARPE. Red arrows indicate the opening of the midpalatal suture. C, D) Clinical photographs, in occlusal view, before and after MARPE, respectively.

The evaluation of axial sections of tomographic images before and after expansion with MARPE evidences opening of the midpalatal suture (Figs 7A and 7B).

## DISCUSSION

Knowing, investigating and discussing the new technologies that appear in the dental market is fundamental to add positive resources to orthodontic diagnosis and planning, which ultimately provide direct benefit for the patients. Concerning the present paper, two questions are important for selection of miniscrews in MARPE, which encouraged the proposal of this systematized protocol. 

**Figure 8:**
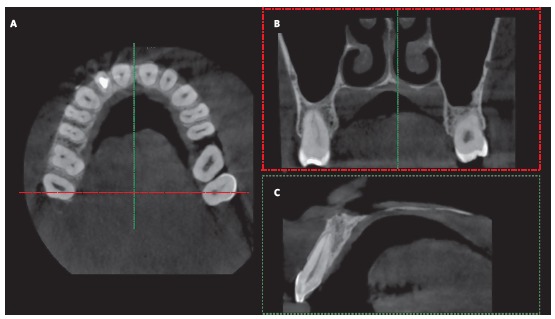
CBCT images. A) Axial section. B) Coronal section at the region of dotted lines (in red, in A). C) Sagittal section at the level of dotted lines (in green, in A), evidencing scarce bone tissue for anchorage of miniscrews in MARPE.

The authors highlight the primary need of anatomical knowledge of the region of interest for MARPE, which may be investigated on cone-beam computed tomographies. After request of CBCT, examining the bone thickness measurement at the area where miniscrews will be inserted aids the diagnosis and prognosis of maxillary expansion. The reduced thickness or lack of bone at the region of miniscrew insertion contraindicate the utilization of the MARPE technique (Fig 8). These cases present little bone anchorage for miniscrews to withstand the load generated by the expanding screw, leading to treatment failure.

The second aspect to be considered in the planning of MARPE refers to the need of establishing the bicortical anchorage of miniscrews to support the expanding screw, on the cortical plates of the oral cavity and nasal fossa, especially at the posterior region of the palate. This region presents greater resistance to opening of the midpalatal suture, yet it presents smaller bone thickness. This occurs due to the complex articulation between palatal bones and maxilla in its anterior region, and sphenoid bone in the posterior region, besides the greater obliteration of sutures in the adult patient.^11^

As previously mentioned, the prognosis of RME in adult individuals is doubtful due to the increased interdigitation of maxillary sutures and rigidity of adjacent structures,^8^ while there has been increased interest by these individuals in the search for orthodontic treatment. Therefore, it is important to combine the knowledge of basic sciences to the evolution of new technologies and therapeutic approaches in the clinic. Studies reporting the effects of RME and more recently of MARPE have been reported in the literature over time.^18^ ^,^ ^23^ ^,^ ^24^ Noticeable results were evidenced in the late treatment of patients (vertebral maturation stages CS4 to CS6)^28^ submitted to MARPE, with positive outcomes in the midpalatal and circummaxillary sutures, pterygopalatine articular structures and nasal cavity, as observed on CBCT images.^11^ Considering these findings, the authors of the present paper observed the need to define a systematized protocol for the selection of miniscrews in the MARPE technique.

In the current market, some companies sell prefabricated devices for MARPE, allowing the orthodontists to fabricate their own expanding screw. However, these appliances present restricted use concerning the adjustment of miniscrews fixation rings height, impairing their utilization in clinical cases with extreme maxillary atresia or palatal asymmetry. Knowing that a great part of patients who may be treated by MARPE presents some of the aforementioned characteristics, the fabrication of expanders by good technicians may overcome such limitations, since the appliance would be customized according to the anatomy of each individual, reducing the risk of failure.

Based on the present protocol for selection of miniscrews for MARPE, it was concluded that it is necessary to know the bone anatomy of the palatal region of interest and midpalatal suture, before the expander miniscrews insertion. This allows the professional greater knowledge for diagnosis, planning and prognosis of maxillary expansion, besides safer application of the MARPE technique. 
